# Analysis of Global Pediatric Cancer Research and Publications

**DOI:** 10.1200/JGO.19.00227

**Published:** 2020-01-02

**Authors:** Eleni Syrimi, Grant Lewison, Richard Sullivan, Pamela Kearns

**Affiliations:** ^1^ University of Birmingham, Institute of Immunology and Immunotherapy, Birmingham, United Kingdom; ^2^ King’s College London, Institute of Cancer Policy, Conflict and Health Research Group, School of Cancer Sciences, Guy’s Hospital, London, United Kingdom; ^3^ University of Birmingham, Cancer Research UK Clinical Trials Unit, National Institute for Health Research (NIHR) Birmingham Biomedical Research Centre, UK Institute of Cancer and Genomic Sciences, Birmingham, United Kingdom

## Abstract

This study sought to investigate the amount of global research activity and investment in pediatric cancer research, using publications as a proxy measure, and to understand geographical differences in research activity. To do this, we used a quantitative method—bibliometrics—to analyze Web of Science publications in the 10 years from 2007 to 2016. We found that global pediatric cancer research outputs have increased from 2,937 in 2007 to 4,513 in 2016, at an annual growth rate of 4.3%. This rate is slower than for both cancer research as a whole and general pediatric research. The increase in output was due almost entirely to China. International collaboration was similar to that in cancer research overall, with the highest levels among countries in close geographical proximity. Hematological and CNS childhood cancers are the main areas for research. Genetics and prognosis were the main research domains, and there was little work on radiotherapy or palliative care. In terms of citations, the best-performing countries were the Netherlands, the United States, and the United Kingdom. On the basis of estimates of the cost of research papers in different countries, the total world pediatric cancer research expenditure is estimated to have been 1.54 billion US dollars (USD) in 2013, and 1.79 billion USD in 2016. Our data suggest that current global policy toward pediatric cancer needs significant review and change to increase investments, balance research portfolios, and improve research that is relevant to low- and middle-income countries.

## INTRODUCTION

Despite the overall improvement in outcomes for most childhood malignancies in high-income settings, cure remains challenging for some diseases, such as bone sarcomas and certain types of brain tumors.^1^ Cancer remains the leading cause of death in children < 15 years old in Western Europe.^2^ Long-term personal and socioeconomic impacts of treatment still remain substantial, with 20%-40% of childhood cancer survivors suffering major long-term disability due to the disease and/or treatment.^3^ In low-income and middle-income countries, chronic infections outweigh cancer deaths in all age groups.^4^ Given the youth bulge in these countries,^5^ there are more cases of childhood cancer than in high-income countries. Survival remains poor because of inadequate treatment or lack of treatment.^6^

Table 1 shows the distribution of the burden (disability-adjusted life years [DALYs], taking into account both early death and years lived with a disability) in 4 groups of countries, classified by income level (high, upper-middle, lower-middle, and low) in 2000 and 2015.^7^ The burden has decreased in upper-middle and high-income countries. However, in low and lower-middle–income countries, the burden has increased.

**TABLE 1:**
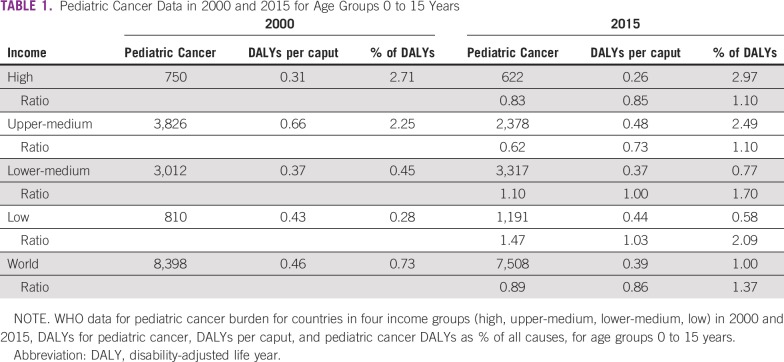
Pediatric Cancer Data in 2000 and 2015 for Age Groups 0 to 15 Years

The importance of research as a major driver for better outcomes has been well recognized and validated.^8^ However, there has been a paucity of strategic intelligence and a tendency to neglect global pediatric cancer research,^9^ making it difficult for policymakers to devise evidence-based strategies. Notably, a 2011 study showed that pediatric cancer accounted for only just over 5% of all cancer research.^10^ Other minor studies examining citations of pediatric cancer papers^11^ and a specific examination of German outputs^12^ added little additional useful data to inform national and international research policies and reflected an overall lack of high-quality intelligence on global pediatric cancer research.

**CONTEXT**

**Key Objective**
What is the state of the global research in the pediatric oncology field?
**Knowledge Generated**
Pediatric cancer research output has been static over the last decade, with a lack of international collaborations. There is a need internationally for greater investment in the field, with increased focus on low- and middle-income settings, where the burden (measured in disability-adjusted life years) from pediatric cancer is much higher.
**Relevance**
Collectively, research and development funds should take these findings into consideration and coordinate initiatives that enhance research and investment in the pediatric cancer field.

In light of the need for an up-to-date and deeper understanding of the state of global research into pediatric cancer, we have used bibliometric methods^13^ to describe the current research activity in this area, particularly changes in the patterns of outputs, the extent of international collaboration, and estimates of global expenditure on pediatric cancer research.

## METHODS

### Selection of Papers

Articles and reviews were selected from the Web of Science (WoS) for the 10 years, 2007-2016, that were identified by both an existing pediatrics filter and a cancer filter. These filters each consisted of lists of specialist journals and title words. They were developed interactively by a bibliometrician (G.L.) and a specialist in the subject area, and their precision (specificity) and recall (sensitivity) were determined by E.S., who marked sets of papers as relevant or not.^14^ The specificity was 0.82 ± 0.024, based on the means of 6 samples of 200 papers. The sensitivity was 0.75, so the filter’s calibration factor (by which the apparent total must be multiplied to give the true” total) was 0.82/0.75 = 1.09.

The bibliographic details of the papers were downloaded, 500 at a time, to a series of text files and then converted to an Excel spreadsheet by means of a visual basic program (macro) written by Philip Roe of Evaluametrics (Saint Albans, United Kingdom). The countries were determined for each paper as fractional counts. For example, a paper with two French addresses and one German one would be classed as FR 0.67, DE 0.33. We focused attention on the leading 16 countries (Table 2).

**TABLE 2:**
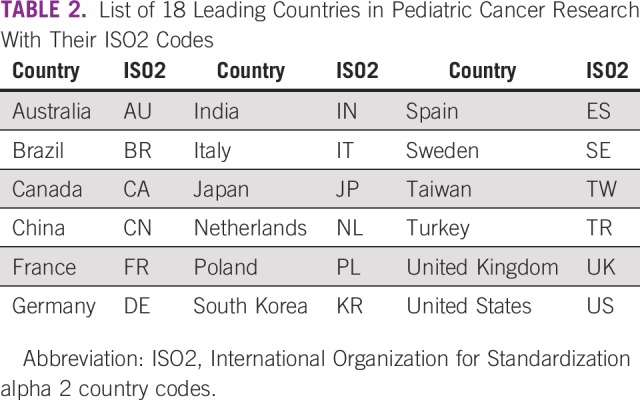
List of 18 Leading Countries in Pediatric Cancer Research With Their ISO2 Codes

### International Collaboration

An overall measure compares the sum of the contributions of individual countries with the world total, which is effectively the sum of the individual country fractional counts, although there are a few papers without addresses. A second measure compares each international pairing with the numbers of coauthored papers that might have been expected if country A chose its partners equally among other countries on the basis of their fractional presence within the field. For example, the foreign contribution to the 2,846 papers with a German author was 902 papers, and of these, Austrian authors contributed 55.5, or 6.15%, whereas their contribution to the field was 246.5 papers on a fractional count basis, or 0.65%. So, their contribution to German papers was more than 9 times what might have been expected (5.9 papers).

### Delineation of Subject Areas

Subfilters were developed by R.S. and G.L., with assistance from Ajay Aggarwal of King’s College London, to identify papers in 10 different research domains and those relevant to 10 individual anatomic cancer sites (Table 3). These also consisted of lists of title words and journal name strings. These subfilters were applied to the pediatric cancer file, and each paper was marked with its research domain(s) and cancer site(s). Some papers could not be classified, and some were classified into more than one domain or site. We also classified the papers by their research level (R.L.) as clinical, basic, or both, on the basis of words in their titles.^15^

**TABLE 3:**
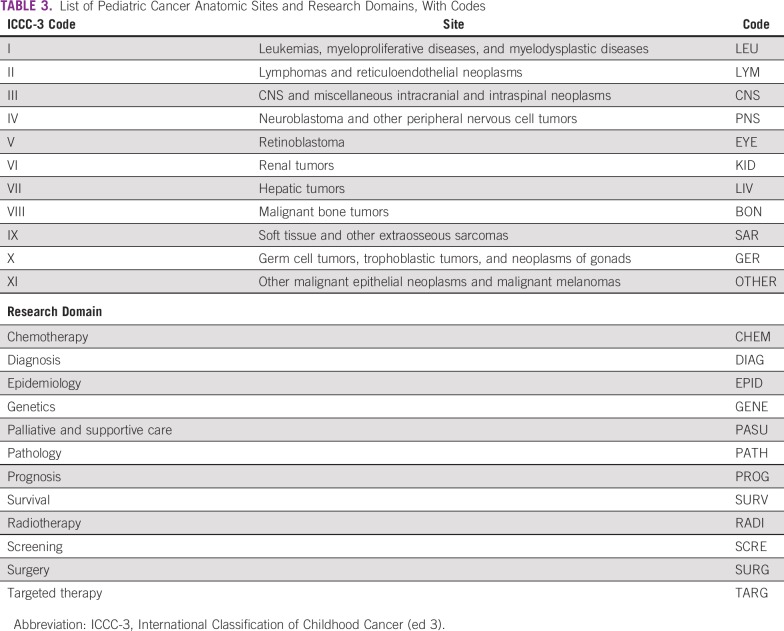
List of Pediatric Cancer Anatomic Sites and Research Domains, With Codes

### Citation Measures

The traditional citation measure for a group of papers is the arithmetic mean of the count of citations in a given time window. This is essential so that all papers are evaluated fairly. We have used a 5-year citation window beginning in the year of publication so as to include the peak year for almost all papers. However, this means our analysis was restricted to the years 2007-2012.

The arithmetic mean of citation counts suffers from bias caused by a few papers having an enormous number of citations, and it has been suggested^16^ that the geometric mean gives a fairer estimate. We augmented the citation counts by unity, then took logarithms of these enhanced counts, weighted them according to each country’s fractional presence on each paper, and then divided the sum of these products by the fractional sum of the country’s citable papers. The resulting quotient was then used as the power of 10 and unity subtracted to give the geometric mean. This was usually approximately half the arithmetic mean.

A third measure of citation performance is the percentage of a country’s papers that are cited highly enough to put them in the top 5% of the total field, or some other percentile. The ratio of this percentage to the nominal percentile, multiplied by 100, is designated the WorldScale value, by analogy with oil tanker charter rates.^17^ For example, Germany published 1,171 citable papers, and 78 of them received enough citations to put them in the top 5%, so its WorldScale value was 100 × 78/(1,171 × 0.05) = 131.

### The Funding of Pediatric Cancer Research

We estimated this by multiplying the estimated cost of a paper in different countries (as a function of income per caput; Data Supplement) by the fractional counts of papers from each country and then summing the products (see Data Supplement for more methodology details.) This gives a more complete tally of expenditures than inquiries of individual funders would do, as there are very large numbers of these, and they may also use different definitions of the subject area and different currencies.

## RESULTS

### Outputs of the World and of Individual Countries

**FIG 1:**
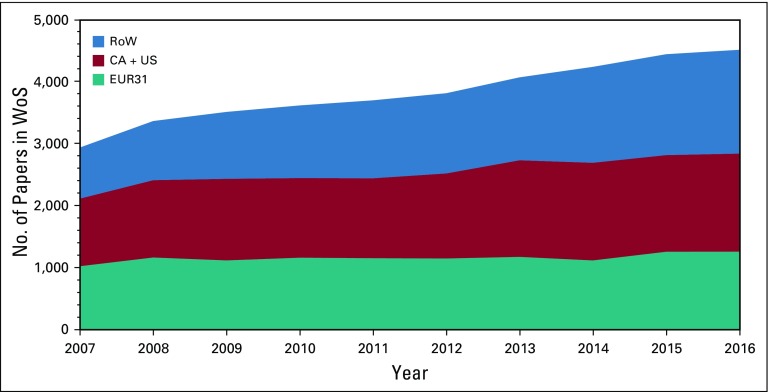
Graph showing outputs of pediatric cancer research papers in the Web of Science (WoS) for 3 groups of countries, 2007-2016. CA, Canada; EUR31, European Economic Area 31; RoW, rest of the world; US, United States.

Figure 1 shows the growth of pediatric cancer research over the 10-year study period 2007-2016 and its division among 3 groups of countries. On average, pediatric cancer research represented 4.7% of all cancer research in 2007-2016. Overall, the number of pediatric cancer research papers increased from 2,937 papers in 2007 to 4,513 in 2016, or by 4.3% per annum (p.a.), but was slower than the growth in cancer research overall or in pediatrics. The proportions of pediatric cancer research within cancer research and pediatrics went down from 5.1% to 4.3% and from 7.8% to 7.2%, respectively.

**FIG 2:**
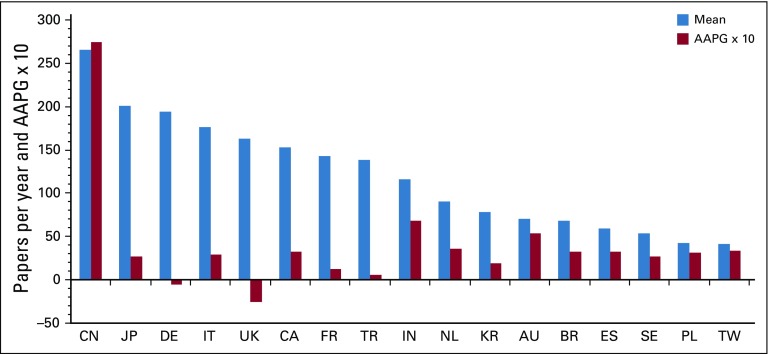
Outputs of pediatric oncology papers per year, 2007-2016, from 17 leading countries, and Annual Average Percentage Growth (AAPG, × 10). AU, Australia; BR, Brazil; CA, Canada; CN, China; DE, Germany; ES, Spain; FR, France; IT, Italy; IN, India; ISO, International Organization for Standardization country codes; JP, Japan; KR, South Korea; NL, Netherlands; PL, Poland; SE, Sweden; TR, Turkey; TW, Taiwan; UK, United Kingdom; US, United States.

The papers were published in 2,831 different journals, but two were used relatively frequently: *Pediatric Blood & Cancer* (3,823 papers, 10.0%) and *Journal of Pediatric Hematology Oncology* (2,100 papers, 5.5%). It was notable that the increase in output from the rest of the world (RoW; Fig 1) is due almost entirely to China, where output grew at 27% p.a. No other country in the top 18 (Data Supplement) exceeded 7% p.a. growth (Fig 2). For two countries, Germany and the United Kingdom, growth was actually negative in this time period. A comparison of country outputs in 2012-2016 with gross domestic product (GDP) is shown in the Data Supplement. The United States and Turkey are clearly outperforming the other countries by a factor of 2. On the other hand, Brazil and Spain are publishing only approximately half of what might have been predicted.

### International Collaboration

There were 38,193 pediatric cancer papers published in 2007-2016, and the sum of the individual country counts was 49,972, or 1.31 times the number of papers. In the same years it was 1.25 for pediatrics research in 2007, increasing to 1.38 in 2016. The corresponding figures for cancer research were 1.24 and 1.35. This shows that the level of international collaboration in pediatric cancer research was similar to that in other fields.

**FIG 3:**
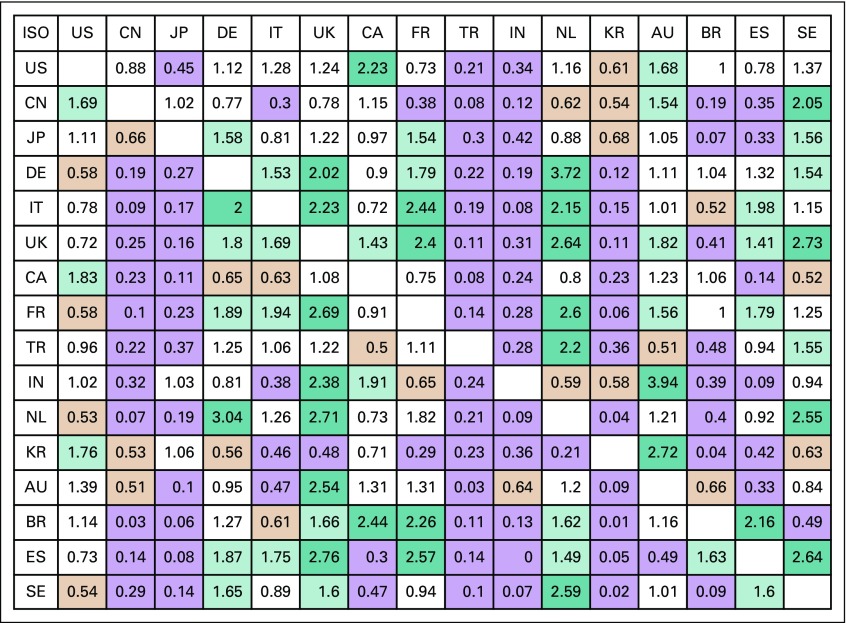
International collaboration expressed as 16 target countries’ (in left column) preference for partnering with other countries (in top row), being the ratio of the observed fractional counts of coauthored papers compared with the counts estimated if they were selected proportionately to their presence in pediatric oncology research (in the absence of papers from the target country). Cells with ratios > 2.0 tinted dark green; with ratios > 1.414 tinted pale green; with ratios < 0.707 tinted orange; with ratios < 0.5 tinted purple. AU, Australia; BR, Brazil; CA, Canada; CN, China; DE, Germany; ES, Spain; FR, France; IT, Italy; IN, India; ISO, International Organization for Standardization country codes; JP, Japan; KR, South Korea; NL, Netherlands; SE, Sweden; TR, Turkey; UK, United Kingdom; US, United States.

Another measure of international collaboration, based on a country’s choice of individual partners, is shown in Figure 3. For many countries, most of the cells show a ratio < 0.05, indicating that they do not favor other countries in this set; this applies in particular to the Asian countries, but there are some exceptions, such as Taiwan’s preference for work with South Korea (data not shown). On the other hand, most European countries prefer to publish with neighboring countries, particularly Germany. Australia chooses partners in India and South Korea much more than might have been expected. However, not all these differences between observed and expected numbers of papers are statistically significant.

### Research Outputs for Different Pediatric Cancers and Research Domains

**FIG 4:**
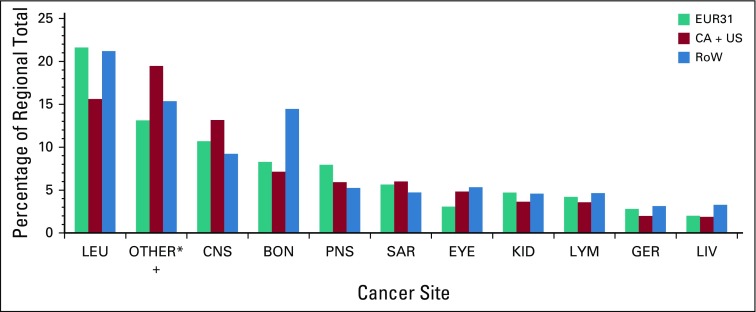
Partition of pediatric oncology research papers by cancer type: from EUR31 countries, Canada, USA, and from the Rest of the World. Percentages of regional totals. BON, malignant bone tumors; CA, Canada; EUR31, European Economic Area 31; GER, germ cell tumors, trophoblastic tumors, and neoplasms of gonads; KID, renal tumors; LEU, leukemias, myeloproliferative diseases, and myelodysplastic diseases; LIV, hepatic tumors; LYM, lymphomas and reticuloendothelial neoplasms; PNS, neuroblastoma and other peripheral nervous cell tumors; RoW, rest of the world; SAR, soft tissue and other extraosseous sarcomas; US, United States. (*) Refers to papers on any other identifiable tumor type as per ICCC-3 Code XI. It also includes papers where the anatomical site is not specified but the title makes clear that they are about pediatric cancer.

**FIG 5:**
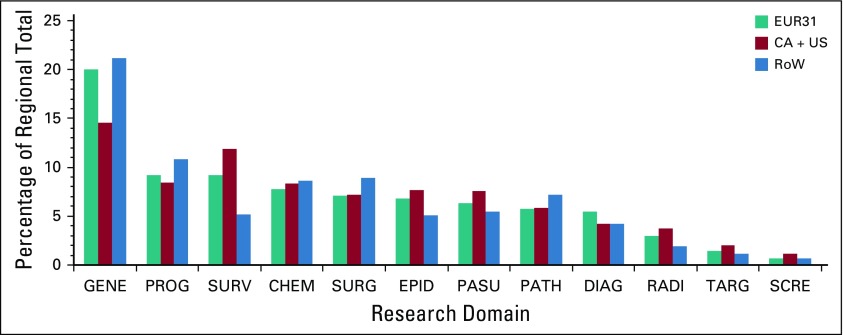
Partition of pediatric oncology research papers by domain (type of research) for papers from European Economic Area 31 (EUR31) countries, from Canada and the United States (CA + US), and from the Rest of the World (RoW). CHEM, chemotherapy; DIAG, diagnosis; EPID, epidemiology; GENE, genetics; PASU, palliative and supportive care; PATH, pathology; PROG, prognosis; RADI, radiotherapy; SCRE, screening; SURG, surgery; SURV, survival; TARG, targeted therapy.

Our results demonstrate that pediatric cancer research was focused on the cancers most prevalent among children, namely, leukemia and CNS cancers (Fig 4). This occurred across all 3 world regions. Examination of the research domains shows that they again were working on broadly similar areas of research (Fig 5). The 2 main treatment modalities in pediatric cancer (chemotherapy and targeted therapy) accounted for 10% of the papers.

A cross-tabulation of the amount of research on the leading cancer types and the 10 research domains showed that the predominant research domains differed between cancer types. As might be expected, surgery is mainly researched in the solid cancers. The ratios of observed to expected totals (integer counts) are shown in the Data Supplement. Another cross-tabulation of the relative commitment of the leading countries and the 3 world regions to different research domains is shown in the Data Supplement.

### Citations of Papers From Leading Countries and in Different Subject Areas

**FIG 6:**
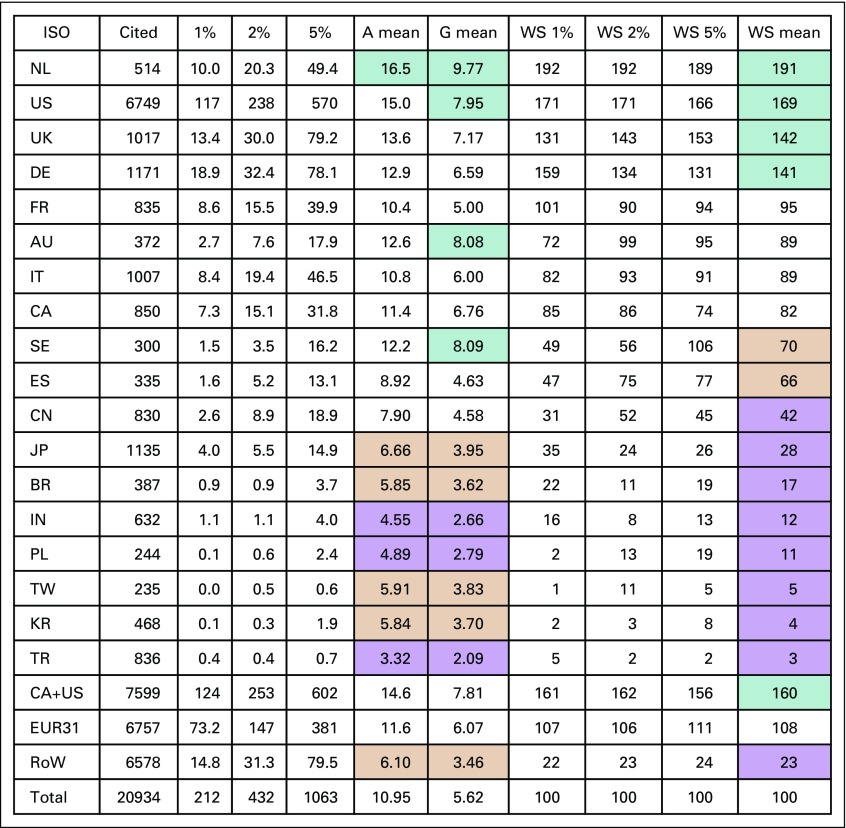
Citation performance of leading 16 countries and 3 world regions in pediatric cancer research, 2007-12, based on their presence in the top centiles, and arithmetic (A) and geometric (G) means of 5-year citation counts. Countries ranked by mean WorldScale score (WS mean). Cells with with ratios > 1.414 world means tinted green; with ratios < 0.707 tinted orange; with ratios < 0.5 tinted purple. AU, Australia; BR, Brazil; CA, Canada; CN, China; DE, Germany; ES, Spain; EUR31, European Economic Area 31; FR, France; IT, Italy; IN, India; ISO, International Organization for Standardization country codes; JP, Japan; KR, South Korea; NL, Netherlands; RoW, rest of the world; SE, Sweden; TR, Turkey; UK, United Kingdom; US, United States.

There were 20,934 potentially citable pediatric cancer papers, and the top 5.08%, or 1,063 papers, received at least 37 citations in the 5-year time window. The results are shown in Figure 6 for the 16 leading countries and for the 3 world regions on 3 different measures. There is a high correlation between these indicators: between the arithmetic and geometric means, *r*^2^ = 0.94; between the arithmetic mean and the mean WorldScale value, *r*^2^ = 0.92; and between the geometric mean and the WorldScale value, *r*^2^ = 0.78. The ranking of the top countries is similar on all 3 indicators, which gives confidence that this is reliable in terms of citation impact. The Netherlands is the clear leader, followed by the United States and the United Kingdom. Almost all (24 out of 27) highly cited papers, with actual citation impact of more than 200 citations, were published in very high-impact journals, with a mean 5-year diachronous citation score (potential citation impact [PCI]) of 154, such as the *New England Journal of Medicine* (PCI = 219) and *The Lancet* (PCI = 181).

### The Funding of Pediatric Cancer Research

**FIG 7:**
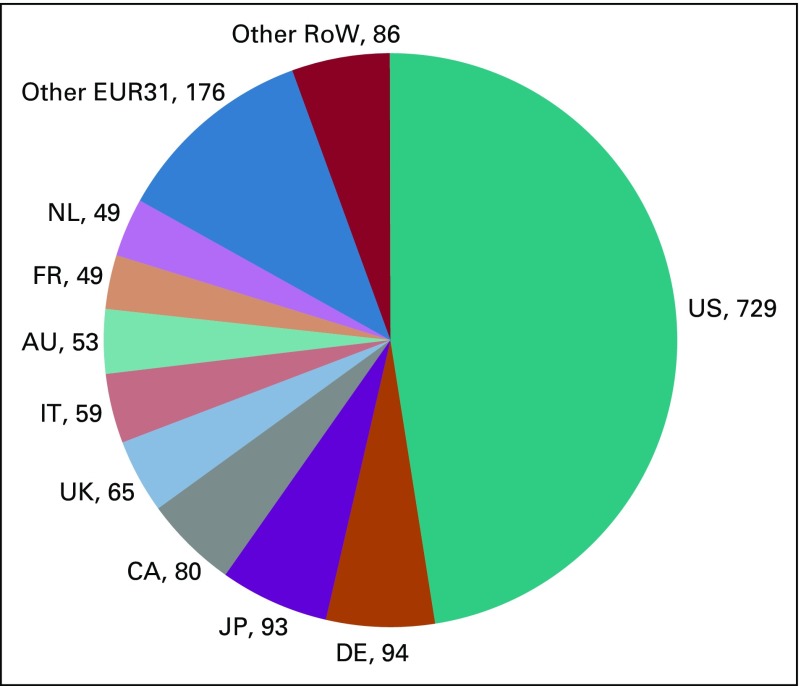
Distribution by country of the estimated world expenditure on pediatric cancer research in 2013, in millions of US dollars, after correction for calibration factor of × 1.09. AU, Australia; CA, Canada; DE, Germany; EUR31, European Economic Area 31; FR, France; IT, Italy; JP, Japan; NL, Netherlands; RoW, rest of the world; UK, United Kingdom; US, United States.

Our estimate of the 2013 costs of a biomedical paper for each country as a function of its wealth per caput is shown in the Data Supplement. These costs, when multiplied by the numbers of papers from each country that year on a fractional count basis, give a pie chart for the global distribution of expenditure (Fig 7). The number of papers has been increased by 9% to account for the lack of recall (0.75) compared with the precision (0.82).

From this analysis, the United States dominated the expenditure even more than it dominated the research output distribution, with nearly 48% of the world total of $1.54 billion in 2013. The next countries, Germany and Japan, each spent just over 6% of the total. Collectively, Canada and the United States spent more than half the total (53%), followed by the EUR31 countries (32%) and the RoW far behind at 15%. Three countries (Japan, Australia, and South Korea) provided 11%, or 71% of the RoW total.

There has been an increase in costs in the United States of approximately 2% per year in the last few years.^18^ When this increment was applied to the cost per paper for each country and to the estimated numbers of papers from each country extrapolated from their totals in 2016, we obtained a total expenditure of $1.79 billion in that year, of which the United States would have contributed $822 million and Japan, the second country, $106 million.

## DISCUSSION

Pediatric cancer research output over the last decade has not kept pace with pediatric and cancer research overall and remains at < 5% of all cancer research output. It is notable that China has made a substantial contribution to the world total, and, without this, global output would have fallen even further. This is a significant issue for both the research community and research funders. However, in the context of national wealth, there appears to be a good correlation between research output and GDP for most countries. On this metric, China’s research output becomes proportionate, while the United States and Turkey are outperforming compared with Brazil, which is underperforming in relation to its GDP. This suggests that countries’ strategies toward pediatric cancer research have become mostly static.

Using publication coauthorship as a surrogate for international collaboration, we saw little difference between the level of collaborative research in pediatric cancer and all types of cancer research. We also noted that there was a tendency for countries to collaborate with those in close geographical proximity. Within Europe, the United Kingdom and the Netherlands were the countries whose research outputs included the highest numbers of collaborators. Given the rarity of pediatric cancers and the existence of well-established international networks, we had expected more collaboration. The data do not give any insights as to what constrains or encourages cross-border collaboration. However, this suggests that despite the efforts of transnational research networks (eg, Innovative Therapeutics for Children with Cancer), current approaches to building and sustaining these networks are not sufficient. This is an issue for both regional funders, such as the European Union, and domestic funding organizations in how they structure and support international collaborations.

The 2 disease areas of cancer research that dominated pediatric cancer were leukemia and CNS, reflecting the prevalence of these disease groups. There was a wide range in the types of research performed across most disease types, with genetics, drug treatments, and prognosis being the favored research domains. There was a notable disease-specific predominance of some research domains, which related to treatment pathway. For example, CNS research was predominantly in radiotherapy and less in other domains. Overall, across most disease areas, there was less research in the fields of palliative and supportive care, targeted therapy, and radiotherapy. There has been a strong demand for more CNS research internationally,^19^ but our data support the need to increase the breadth of pediatric cancer research to include other areas of unmet clinical and research need.

Although the outputs of papers from the 3 world regions are quite similar (Fig 1), the burden measured in DALYs^20^ from pediatric cancers is much higher in the RoW than in Europe or North America. This speaks to one of the most critical and growing schisms in global pediatric cancer research, namely, that between high-resource and middle-low–resource settings. This is a major failure that requires urgent review by the leading research funders and overseas development funds. Moreover, our finding about the hegemonic domination of research by high-income countries is an issue that needs urgent attention.

The number of times a paper has been cited reflects the impact of research produced on other researchers.^21^ The Netherlands, the United States, and the United Kingdom are the countries with the most frequently cited papers. The Netherlands spent only 3.2% of the world total in 2013, but its papers were the most cited. Although this reflects well on Dutch research, our analysis shows the need to increase context-relevant research from low-middle–income countries with a growing burden from pediatric cancer.

Our study results are limited because we wanted to include as many papers as possible to gain a better understanding of global pediatric cancer research output, but this introduced a potential error in papers included. The precision of the pediatric cancer filter was 82%, which means that nearly one-fifth of the papers may have been included in error. However, as the recall was less than this (0.75), the number of papers was actually underestimated by approximately 9%. Finally, our estimates of the expenditure on pediatric cancer research depend on the assumption that the average cost of a paper in this field is similar to that in other noncommunicable diseases research and that the cost does not vary greatly by research domain or anatomic site. The estimates also neglect those intramural expenditures by companies that do not lead to publications in the open literature,

As reflected in the WHO Global Childhood Cancer Initiative,^22^ there is increasing recognition of the importance of addressing the needs of childhood cancer, and research is central to improving patient care and outcome. Our data suggest there is a need internationally for greater investment in this field. Moreover, the current international networks are being underused for cross-border collective research. Collectively, the multidisciplinary stakeholders in childhood cancer research would be well placed for coordinated initiatives that ensure the full breadth of childhood cancer needs that are addressed in their respective research programs.
